# Morphological changes of large layer V pyramidal neurons in cortical motor-related areas after spinal cord injury in macaque monkeys

**DOI:** 10.1038/s41598-022-26931-3

**Published:** 2023-01-03

**Authors:** Yu Takata, Hajime Yamanaka, Hiroshi Nakagawa, Masahiko Takada

**Affiliations:** 1 Primate Research Institute and Center for the Evolutionary Origins of Human Behavior, Kyoto University, Inuyama, Aichi 484-8506 Japan; 2 Department of Molecular Neuroscience, World Premier International Immunology Frontier Research Center, Osaka University, Suita, Osaka 565-0871 Japan; 3 Systems Neuroscience Section, Primate Research Institute, Kyoto University, Inuyama, Aichi 484-8506 Japan; 4 Present Address: Okinawa Institute of Science and Technology Graduate University, Kunigami, Okinawa 904-0495 Japan

**Keywords:** Neuroscience, Neurology

## Abstract

In primates, neurons giving rise to the corticospinal tract (CST) are distributed in several motor-related areas of the frontal lobe, such as the primary motor cortex (M1), the supplementary motor area (SMA), and the dorsal and ventral divisions of the premotor cortex (PMd, PMv). Recently, we have shown in macaque monkeys that the morphology of basal dendrites of CST neurons, i.e., large layer V pyramidal neurons, varies among the digit regions of the motor-related areas. Here, we investigated the alterations in basal dendrite morphology of CST neurons after spinal cord injury (SCI). In our monkey model, both the complexity and the spine density of basal dendrites were highly decreased throughout the areas. Notably, these events were less prominent for the PMd than for the M1, SMA, and PMv. In analyzing the density changes post-SCI of the filopodia-, thin-, stubby-, and mushroom-type spines, it was found that the density of filopodia-type spines was increased for all areas, whereas the other types of spines exhibited density decreases. Such spine density reductions were so limited for the PMd as compared to the other areas. The observed plastic changes of CST neurons may contribute to the recovery from impaired motor functions caused by SCI.

## Introduction

Neurons communicate with each other through synapses, formed on postsynaptic dendrites and dendritic spines and generate complex neural networks that enable various modes of information processing. In the cerebral cortex, pyramidal neurons are the principal excitatory neurons responsible for network signal transmission^1^. The pyramidal neurons generally possess apical and basal dendrites^2^ with enriched dendritic spines which can be classified into five types by shape, i.e., filopodia, thin, stubby, mushroom, and branched types^3^. It has been well documented that the complexity of dendrites and the morphology of dendritic spines dramatically alter not only in developmental and learning processes, but also after nerve injury^4–6^. Previous studies reported that in rodent models of stroke and spinal cord injury (SCI), pyramidal neurons in the sensorimotor cortex were subjected to morphological changes^7,8^. According to these studies, the complexity of basal dendrites of the pyramidal neurons and the density of their spines were decreased after the lesioning.

It is well known that the corticospinal tract (CST) is the major pathway conveying cortically-processed motor commands directly to the spinal cord^9,10^. Although the CST is commonly present in most mammals, its architecture varies among species. In fact, there exist marked differences between rodents and primates in the route and spinal termination of CST fibers and the cortical distribution of CST neurons^11^. The main component of long-descending CST fibers reaches spinal interneurons and/or motoneurons. In rodents, the CST fibers pass through the dorsal funiculus and terminate primarily on spinal interneurons (including propriospinal neurons) to connect to spinal motoneurons^12^. In primates, on the other hand, the CST fibers travel mostly through the lateral funiculus contralateral to the hemisphere while partly descending in the ipsilateral lateral funiculus and in the ventral funiculus, and at least some of them terminate directly on spinal motoneurons^13^.

With respect to the cells of origin of the CST, primate CST neurons are composed of large pyramidal neurons in layer V of the frontal motor-related areas, such as the primary motor cortex (M1), the supplementary motor area (SMA), and the dorsal and ventral divisions of the premotor cortex (PMd, PMv)^14–17^. Recently, we have demonstrated in macaque monkeys that both the basal dendrite complexity and the dendritic spine density of large layer V pyramidal neurons, i.e., putative CST neurons, differ among the digit regions of the motor-related areas^18^. Since basal dendrites of pyramidal neurons generally receive more excitatory inputs than apical dendrites^19^, their morphological changes are likely to play an important role in neural plasticity after nerve injury as well as during development/learning^20^. However, it remains obscure how the extent of alterations in the morphology of CST neurons following SCI may be different from area to area. Using a primate model of SCI, we therefore investigated and compared the patterns of changes in the arborization of basal dendrites and the density of their spines of putative CST neurons distributed in the digit regions of individual motor-related areas of the frontal lobe.

## Results

In the present study, two Japanese macaques (Monkeys A and B) were used as a normal control, and the other two macaques (Monkeys C and D) were as an SCI model. These monkeys were trained a reaching and grasping task. In parallel with the task training, intracortical microstimulation (ICMS) mapping was performed to identify the digit regions of the M1, SMA, PMd, and PMv. In Monkeys C and D, the spinal cord was lesioned at the level of the cervical enlargement following 5-day behavioral evaluation. For 7 days after SCI, the monkeys were allowed to recover general conditions. Then, the monkeys again underwent electrophysiological (with ICMS) and behavioral evaluation. Three days later (10 days after SCI), the monkeys were sacrificed for morphological analyses. The above time course of our experiments is shown as Supplementary Fig. 1a.

### ICMS mapping

**Figure 1:**
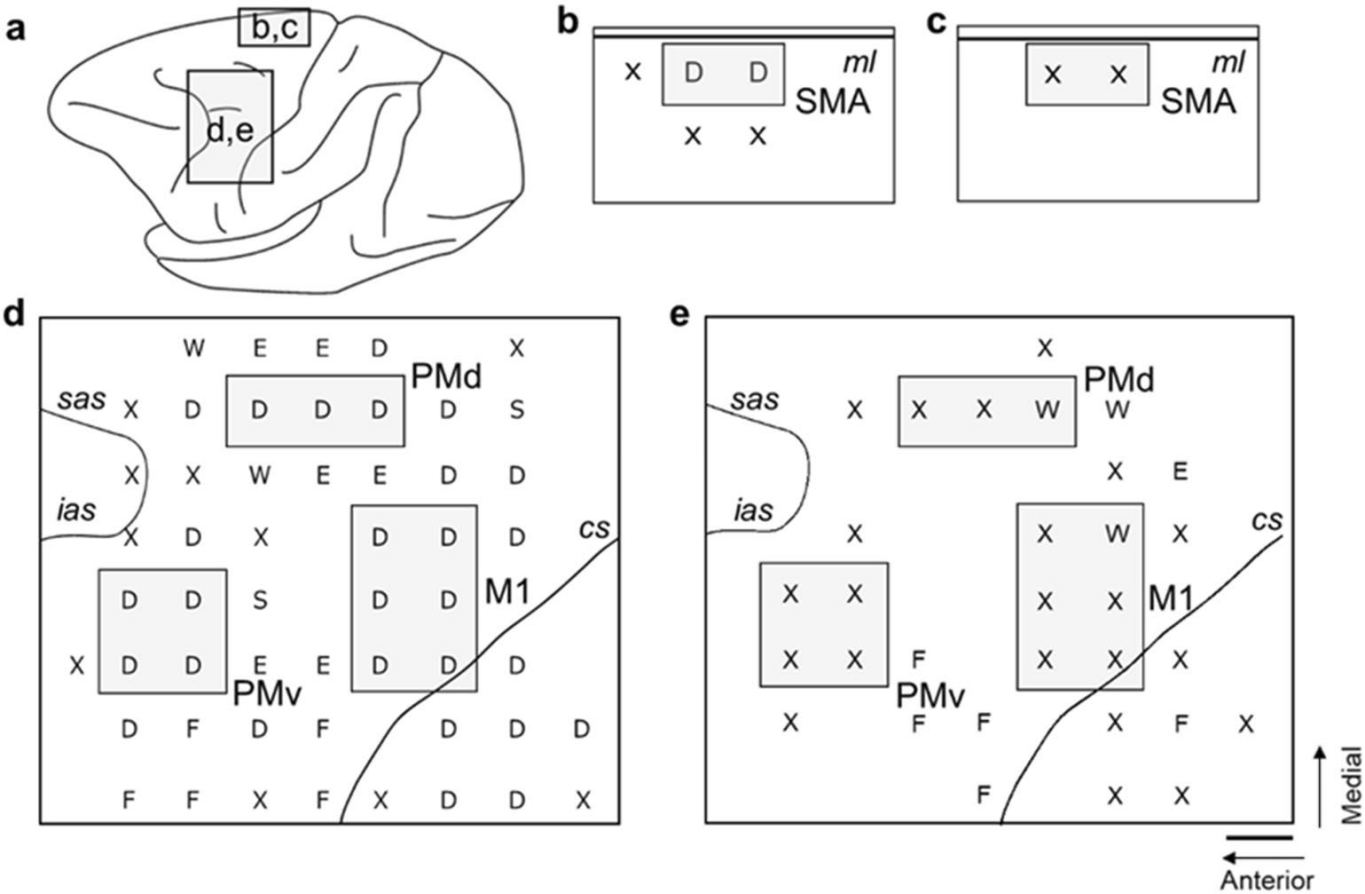
ICMS mapping of frontal motor-related areas in a monkey SCI model. (**a**) Lateral view of the monkey brain showing the cortical areas examined by ICMS. (**b**,**c**) Results of ICMS mapping for identification of digit representation of the SMA before SCI (**b**) and 10 days after SCI (**c**) in Monkey C. (**d**,**e**) Results of ICMS mapping for identification of digit representations of the M1, PMd, and PMv before SCI (**d**) and 10 days after SCI (**e**). Scale bar, 2 mm. The identified digit regions (in gray) are taken for morphological analyses of large layer V pyramidal neurons (i.e., putative CST neurons). The body parts of which movements were evoked by ICMS are indicated as follows: D, digits; E, elbow; F, face; S, shoulder; W, wrist; X, no response. cs, central sulcus; ias, inferior limb of the arcuate sulcus; ml, midline; sas, superior limb of the arcuate sulcus.

In the present study, it was critical to isolate the digit region in each motor-related area as accurately as possible. We carried out ICMS to identify the digit regions in individual motor-related areas (Fig. 1a). Using the same ICMS technique, we further confirmed motor impairments therein after SCI. In the normal control, movements of the digits were observed on ICMS in each motor-related area. Prior to SCI, digit movements were evoked from six or more loci within the M1 (including both the anterior bank of the central sulcus, new M1 and the convexity, old M1^21^), two loci within the SMA, three or more loci within the PMd, and four or more loci within the PMv (Fig. 1b,d). Following SCI, however, no such movements were elicited by ICMS in each area (Fig. 1c,e). Based on the result of ICMS mapping before SCI, a discrete digit region in each motor-related area was dissected out in a tissue block for morphological analyses of large layer V pyramidal neurons (i.e., putative CST neurons).

### SCI model

In the two monkeys, the cervical enlargement of the spinal cord was unilaterally lesioned at the border between the C6 and the C7 segment (Suppl. Fig. 1b). The CST fibers travelling through the dorsolateral funiculus were completely removed, leaving the ventral funiculus intact (Suppl. Fig. 1c).

### Behavioral analysis

**Figure 2:**
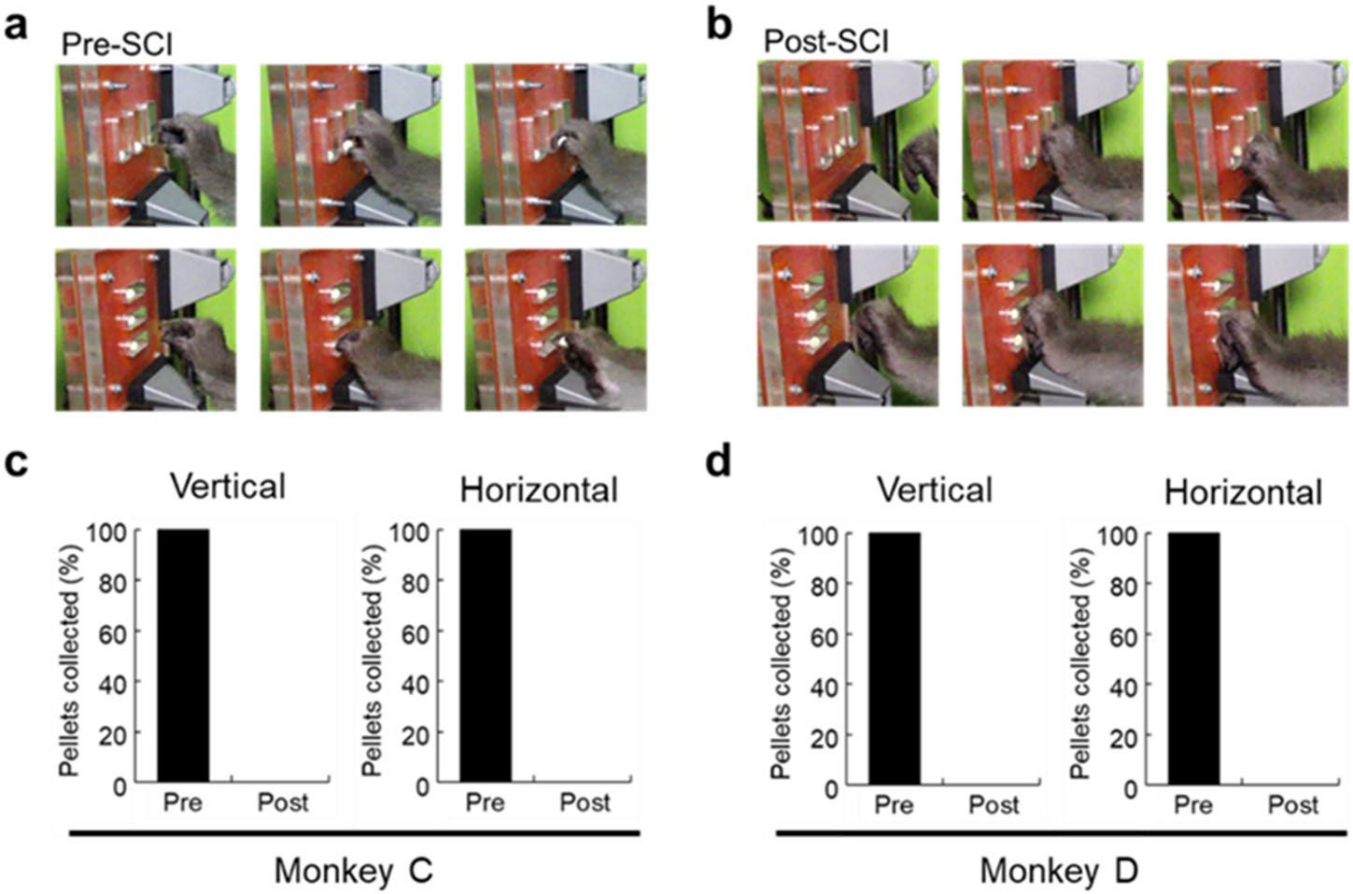
Behavioral evaluation of manual dexterity. (**a**,**b**) Representative frames showing the performance of a reaching and grasping task. The monkey is required to reach a vertical or horizontal slot and grasp a pellet within 10 s. In (**a**) and (**b**), dexterous movements before SCI (Pre-SCI) and 10 days after SCI (Post-SCI) are depicted, respectively. (**c**,**d**) Ratios of pellets collected from vertical or horizontal slots before (Pre) and after (Post) SCI in Monkeys C and D. These monkeys could reach the slots, but not grasp the pellets.

To assess the extent to which manual dexterity was impaired by SCI, behavioral analysis using a reaching and grasping task was performed for 5 days pre-SCI and on the 10th day post-SCI. After SCI, the monkeys could no longer collect pellets at all from vertical or horizontal slots (Fig. 2).

### Morphological analyses of large layer V pyramidal neurons

In our recent study^18^, we morphologically identified CST neurons by retrograde labeling from the cervical enlargement, especially the C6–T1 segments responsible for digit innervation and measured the somal size of labeled CST neurons in the motor-related areas. According to our analysis, the somal size of CST neurons in each area was defined as larger than 238.29 µm^2^ for the M1, 221.49 µm^2^ for the SMA, 199.78 µm^2^ for the PMd, and 179.6 µm^2^ for the PMv. In the present work, 20 large pyramidal neurons with their somal size that met the above criteria of CST neurons were selected from layer V of the digit regions of individual motor-related areas as follows: 445.82 ± 20.63 µm^2^ for the M1, 438.87 ± 17.21 µm^2^ for the SMA, 283.17 ± 5.91 µm^2^ for the PMd, 314.58 ± 11.63 µm^2^ for the PMv.

**Figure 3:**
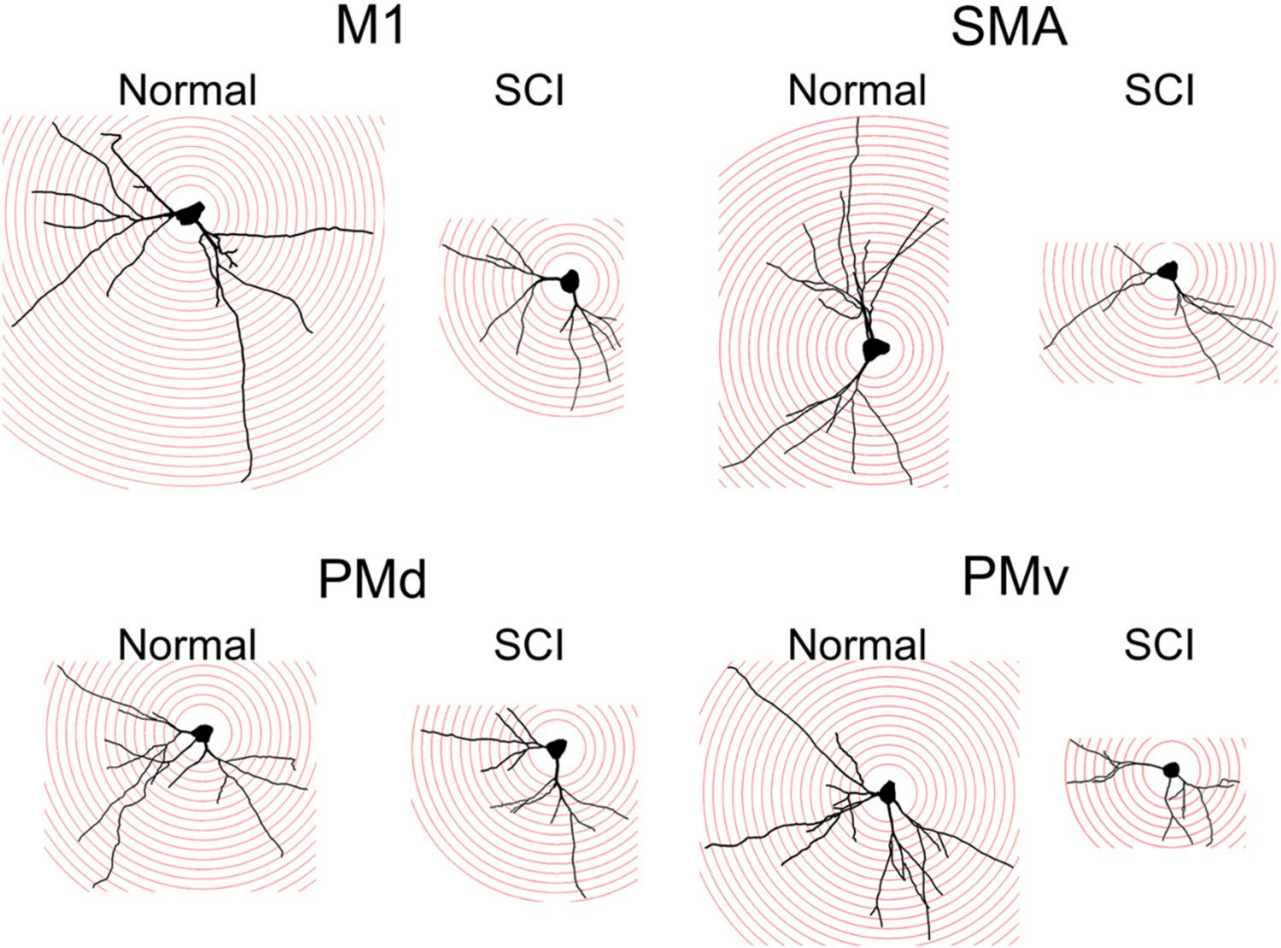
Arborization of basal dendrites of large layer V pyramidal neurons in frontal motor-related areas in a normal and an SCI model. Drawings of basal dendrites of large layer V pyramidal neurons in the M1, SMA, PMd, and PMv in a normal control and an SCI model. For Sholl analysis, concentric circles were utilized starting at 30 µm away from the center of soma and increasing radii by 10 µm.

**Figure 4:**
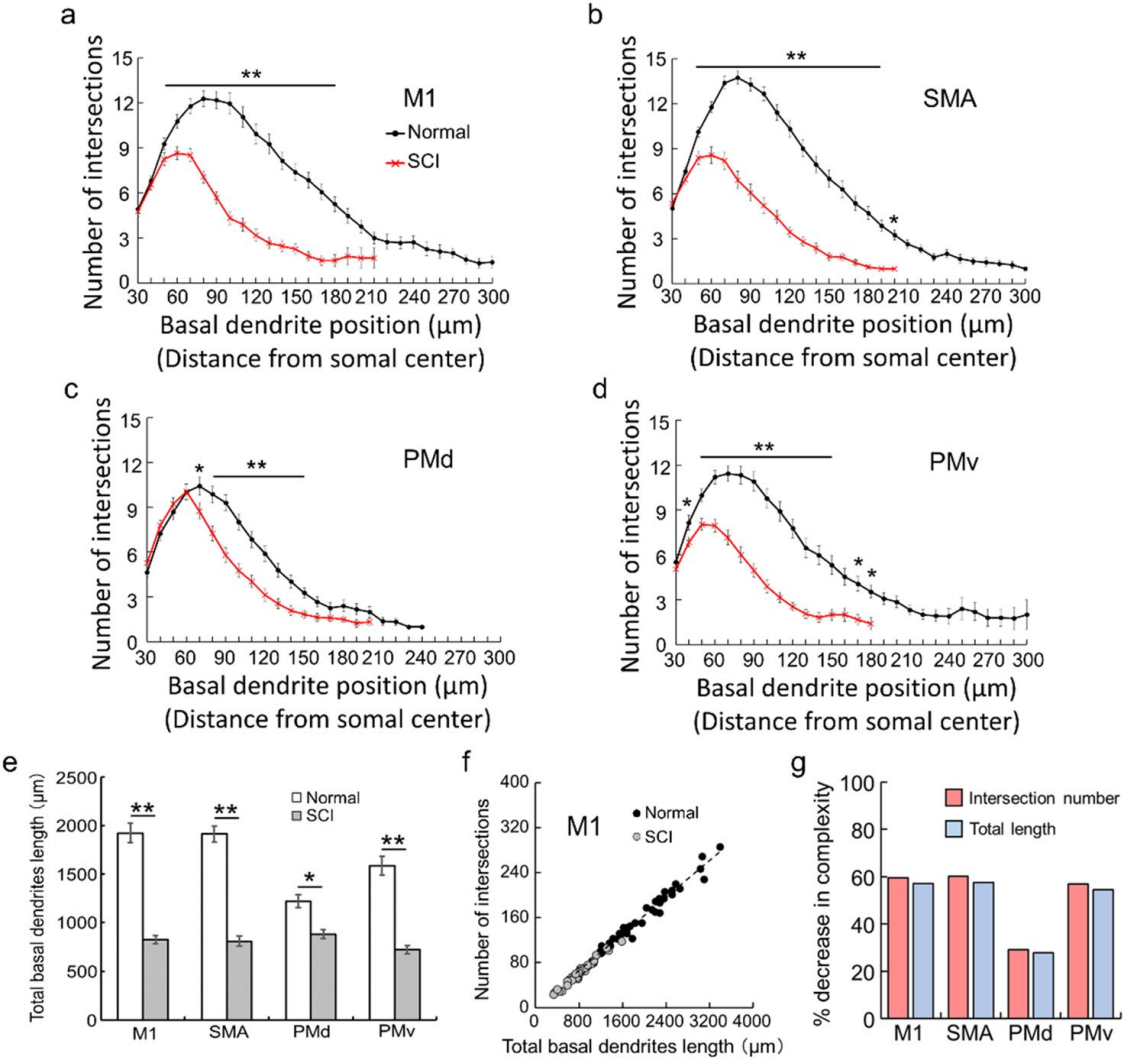
Reduced arborization post-SCI of basal dendrites of large layer V pyramidal neurons in frontal motor-related areas. (**a**–**d**) Histograms showing the number of intersections of basal dendrites at every 10-µm position for the M1 (**a**), SMA (**b**), PMd (**c**), and PMv (**d**) in the normal control (in black) and SCI model (in red). Error bars denote SEM. Two-way ANOVA with the Tukey–Kramer method. *p < 0.05, **p < 0.01. (**e**) Histogram showing the total length of basal dendrites in the normal control (in white) and SCI model (in gray). (**f**) Correlations between the intersection number and the total length of basal dendrites in the M1 in the normal control (in black) and SCI model (in gray). (**g**) Histogram showing the decrease ratios of intersection number (in red) and total length (in blue) after SCI.

The basal dendrites of these neurons were successfully traced, as the full length of each dendrite could be followed completely in single sections. For individual motor-related areas, the complexity of basal dendrites, consisting of the number of intersections and the total length of basal dendrites, was analyzed by means of Sholl analysis (Fig. 3; for details, see “Materials and methods”). In the SCI model, the complexity of basal dendrites for each motor-related area became simpler than in the normal control (TukeyHSD; *p* < 0.05); the intersection number became smaller and the total basal dendrites length became shorter (Fig. 4a–e). These two values were strongly correlated with each other in both the normal and the SCI cases (For the M1, see Fig. 4f). For the M1, SMA, and PMv, the complexity of basal dendrites in the SCI model was decreased by about 60% in comparison with the normal control. For the PMd, on the other hand, it was decreased by less than 30% (Fig. 4g).

**Figure 5:**
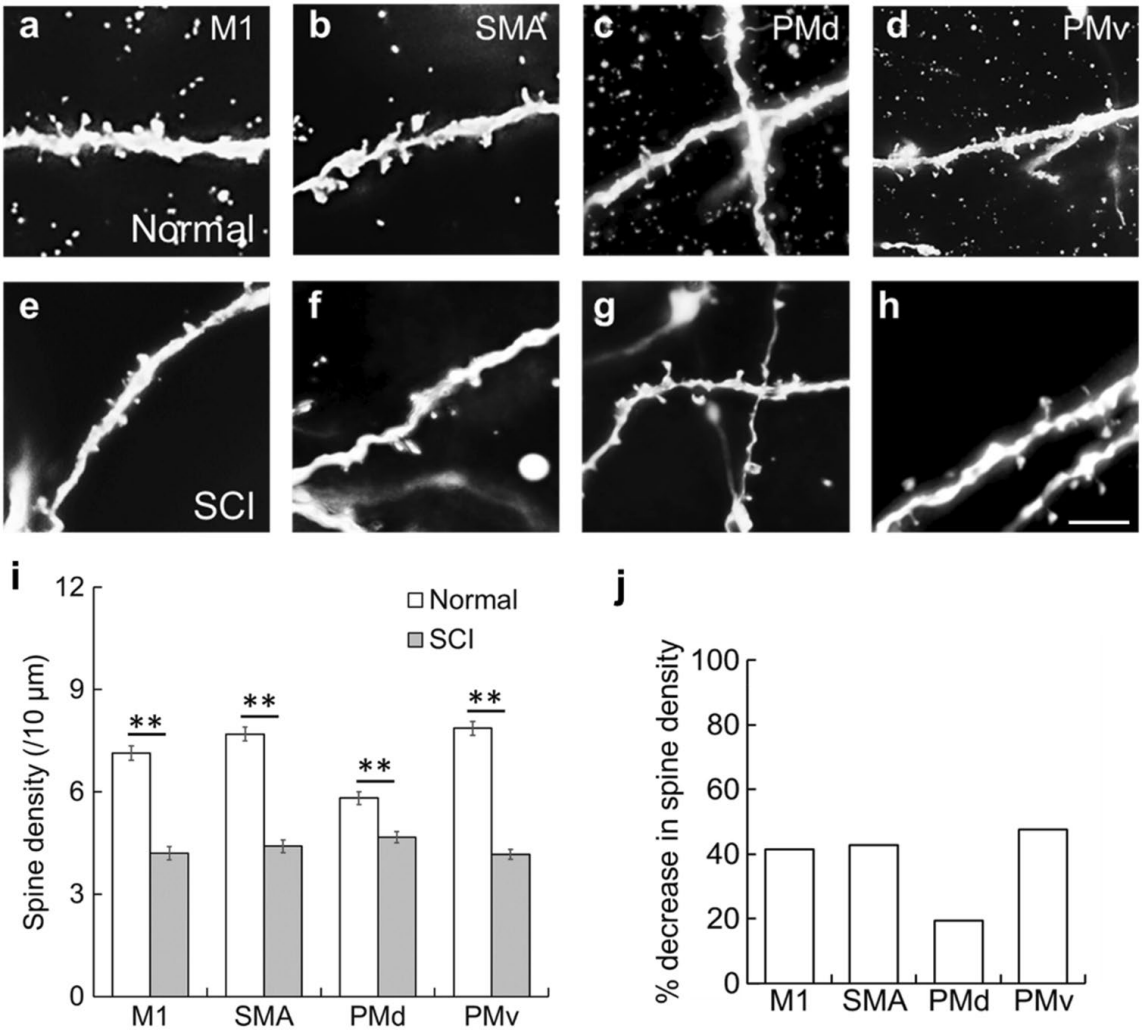
Decreased spine density post-SCI of basal dendrites of large layer V pyramidal neurons in frontal motor-related areas. (**a**–**h**) Representative morphology of spines on basal dendrite(s) of large layer V pyramidal neurons in the M1 (**a**,**e**), SMA (**b**,**f**), PMd (**c**,**g**), and PMv (**d**,**h**) in a normal control (**a**–**d**) and an SCI model (**e**–**h**). Scale bar, 4 µm. (**i**) Histogram showing the spine density of basal dendrites in the normal control (in white) and SCI model (in gray). Expressed as the number of spines per 10-µm segment of single representative dendrites (n = 20). Error bars denote SEM. Two-way ANOVA with the Bonferroni post hoc test. **p < 0.01. (**j**) Histogram showing the decrease ratios of spine density after SCI.

Subsequently, the density of dendritic spines on single basal dendrites was compared between normal control and SCI model monkeys. For each motor-related area, the dendritic spine density in the SCI model was lower than in the normal control (Two-way ANOVA with the Bonferroni post hoc test; *p* < 0.01; Fig. 5a–i). The density for the M1, SMA, and PMv was decreased by about 40% in the SCI model as compared to the normal control. On the other hand, the dendritic spine density for the PMd was decreased by less than 20% after SCI (Fig. 5j).

**Figure 6:**
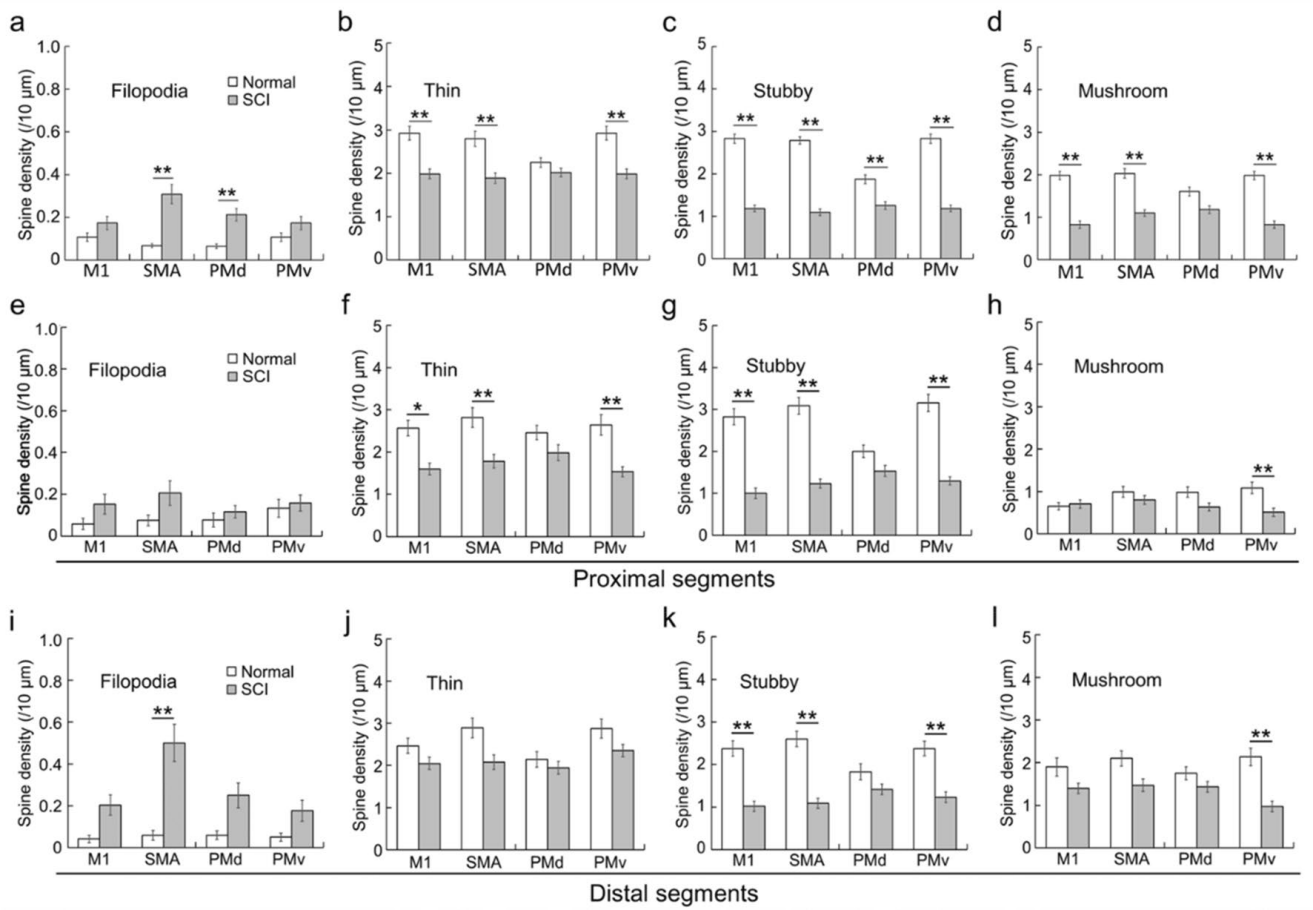
Density changes post-SCI of filopodia-, thin-, stubby-, and mushroom-type spines of basal dendrites of large layer V pyramidal neurons in frontal motor-related areas. (**a**) Increase in the density of filopodia-type spines. (**b**–**d**) Decreases in the density of thin-, stubby-, and mushroom-type spines. (**e**–**l**) Alterations in the density of filopodia-, thin-, stubby-, and mushroom-type spines on the proximal (30–60 µm from the dendritic origin; (**e**–**h**) and distal (30 µm from the dendritic tip; (**i**–**l**) segments of basal dendrites. Two-way ANOVA with the Bonferroni post hoc test. *p < 0.05, **p < 0.01.

We then compared the density of each of the five spine types, i.e., filopodia, thin, stubby, mushroom, and branched types (for details, see “Materials and methods”), on single basal dendrites in the SCI model with that in the normal control. Since the branched type was only rarely observed for individual motor-related areas, it was precluded from analysis. For the SMA and PMd, the density of filopodia-type spines became higher in the SCI model, albeit their absolute number was quite small (Two-way ANOVA with the Bonferroni post hoc test; *p* < 0.01; Fig. 6a). No significant difference was detected for the M1 and PMv, though the filopodia-type spine density tended to be higher compared with the normal control (Fig. 6a). With respect to the other three types of spines, their distributions were negatively changed after SCI. For the M1, SMA, and PMv, the density of each of the thin-, stubby-, and mushroom-type spines was lower in the SCI model than in the normal control (Two-way ANOVA with the Bonferroni post hoc test; *p* < 0.01; Fig. 6b–d). For the PMd, on the other hand, there was no significant difference in the density of thin- and mushroom-type spines, while stubby-type spines displayed a lower density in the SCI model than in the normal control (Two-way ANOVA with the Bonferroni post hoc test; *p* < 0.01; Fig. 6b–d). Concerning all these spine types, their density reductions were so limited for the PMd as compared to the other motor-related areas (Fig. 6b–d).

We further analyzed the distribution patterns of filopodia-, thin-, stubby-, and mushroom-type spines for individual motor-related areas in the control and SCI model. When the basal dendrites were divided into proximal and distal segments, it was found that the increased density of filopodia-type spines post-SCI was more marked in the distal segments (Fig. 6e,i), whereas the density of each of thin- and stubby-type spines was more prominent in the proximal segments (Fig. 6f,g,j,k). Consistent with the data shown in Fig. 6b–d, the decrease in thin-, stubby-, or mushroom-type spine density was limited for the PMd, especially in the distal segments of basal dendrites (Fig. 6f–h,j–l).

**Figure 7:**
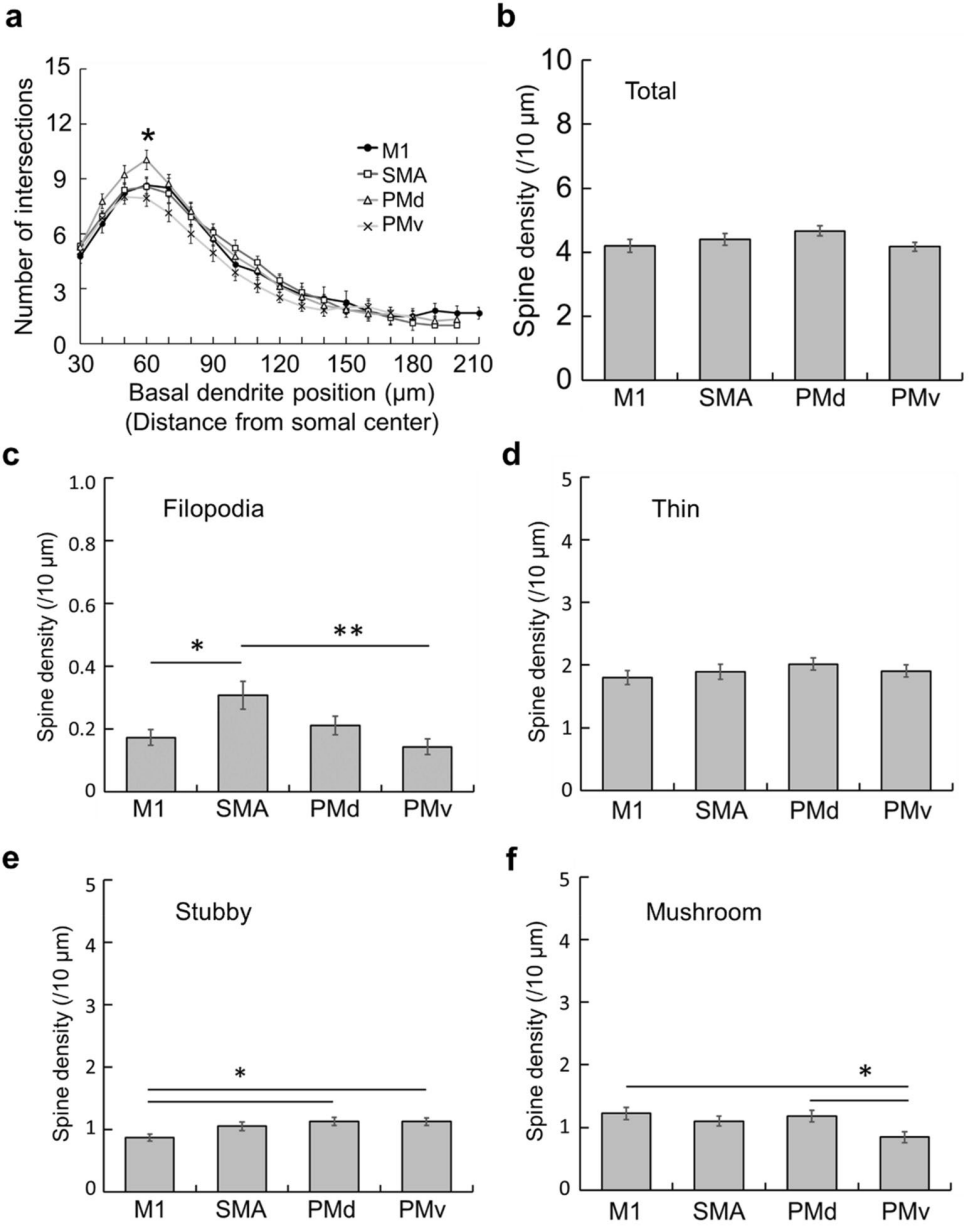
Morphological changes post-SCI of basal dendrites of large layer V pyramidal neurons in frontal motor-related areas. (**a**) Histogram showing the number of intersections of basal dendrites at every 10-µm position for the M1, SMA, PMd, and PMv. Two-way ANOVA with the Tukey–Kramer method. *p < 0.05. (**b**–**f**) Histograms showing the density of total spines (**b**), and filopodia- (**c**), thin- (**d**), stubby- (**e**), and mushroom-type (**f**) spines of basal dendrites for the M1, SMA, PMd, and PMv. One-way ANOVA with the Tukey–Kramer method. *p < 0.05, **p < 0.01.

When the morphological alterations of basal dendrites after SCI were compared among the motor-related areas, we confirmed that the number of intersections through the dendrites was similar across the areas, except that it tended to be larger or smaller at quite proximal sites for the PMd or PMv, respectively (Fig. 7a). Also, there were relatively little differences in the dendritic spine density among the areas, though some significant differences were seen in filopodia-, stubby-, and mushroom-type spines (Fig. 7b–f).

**Figure 8:**
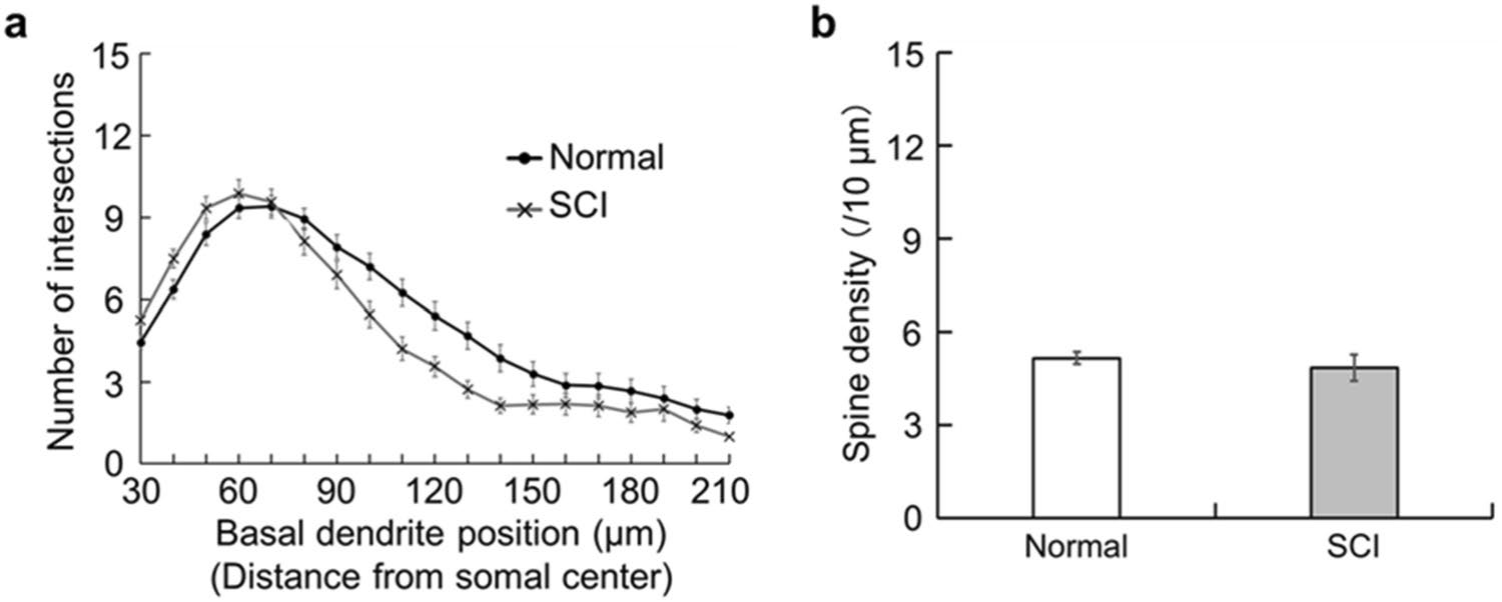
Morphology of basal dendrites of large layer V pyramidal neurons in M1 face region. (**a**) Histogram showing the intersection number of basal dendrites at every 10-µm position in a normal control (Normal) and an SCI model (SCI). Two-way ANOVA. (**b**) Histogram showing the spine density of basal dendrites in a normal control (in white) and an SCI model (in gray). Student t-test.

Finally, the morphology of basal dendrites of large layer V pyramidal neurons (somal size, 306.32 ± 18.18 µm^2^) in the face region of the M1 was analyzed in the normal control and SCI model. In comparison with the normal control, no significant change in either the intersection number or the spine density of the dendrites was detected in the SCI model (Fig. 8).

## Discussion

Using a primate model of SCI, we analyzed the morphology of putative CST neurons in the frontal motor-related areas to reveal the structural changes in basal dendrites. In our model, the CST fibers travelling through the dorsolateral funiculus were totally removed at the border between the C6 and the C7 segment. However, the ventral funiculus containing the anterior CST was spared to innervate both the contralateral and the ipsilateral spinal segments^13^. It should also be mentioned that some descending axons in the dorsolateral CST may cross the midline below the C7 segment to reach the spinal cord on the lesioned side. In this case, it is possible that the putative CST neurons examined in the present study likely contain neurons whose axons escaped incision.

Previous studies in mice have shown that the morphology of CST neurons in the sensorimotor cortex is altered following SCI^8,22^. Kim et al.^8^ have reported that the density of dendritic spines was decreased within 1 week after SCI and gradually recovered by 4 weeks. It has also been suggested that the spine density decrease in the acute phase post-SCI may be due to the reduced CST neuron activity. On the other hand, Ghosh et al.^22^ have demonstrated that the decrease in spine density after SCI was larger in proximal apical dendrites than in distal apical dendrites of CST neurons, which might reflect reduced input from the neighboring neurons. It is generally accepted that CST neurons in primates, including humans, are widely distributed over multiple motor-related areas, such as the M1, SMA, PMd, and PMv, which are structurally and functionally differentiated within the frontal lobe^15,16,23–25^ However, no data have so far been available on morphological changes post-SCI of CST neurons in these motor-related areas. It can be considered that such alterations in CST neuron morphology are differentially organized across the areas. Recently, we have reported that the anatomical features of basal dendrites of putative CST neurons (i.e., large layer V pyramidal neurons) vary among individual areas, especially their digit regions, in normal macaque monkeys^18^. Of particular interest was that based on the total length and intersection number, the basal dendrites of putative CST neurons were more poorly developed for the PMd compared with the M1, SMA, and PMv. Further, single basal dendrites possessed spines less densely for the PMd than for the other motor-related areas. While it remains to be elucidated exactly how these findings reflect the functional and/or hodological characteristics of CST neurons in the motor-related areas, their morphological differences may possibly be attributed to structural changes during the development of individual areas^4,26^.

Employing a primate model of SCI, we have revealed the morphological alterations of basal dendrites of putative CST neurons in the frontal motor-related areas. Here we analyzed the complexity of the dendrites and the dendritic spine density in terms of distinct spine types classified according to their maturity. In our study, the SCI model in which CST fibers were lesioned between the C6 and the C7 segment was prepared in macaque monkeys, given that the spinal motoneurons innervating the digit muscles are located below the C7 segment^27,28^. To assess the extent of impaired digit movements, we performed behavioral analysis using the reaching and grasping task, and electrophysiological examination by means of ICMS. Then, we confirmed that digit movements were severely impaired both behaviorally and electrophysiologically. In this SCI model, each motor-related area exhibited the reductions in both the complexity and the spine density of basal dendrites of putative CST neurons. These morphological changes are ascribable to SCI, because no discernible changes in basal dendrite morphology were observed in large layer V pyramidal neurons located in the face region of the M1. Notably, such dendritic events in response to SCI were less prominent (by about half) for the PMd than for the M1, SMA, and PMv. The differential changes in basal dendrite morphology among the motor-related areas may be attributed to the difference in activity of large layer V pyramidal neurons in each of the motor-related areas after SCI. It should be noted here that Kim et al.^8^ have reported that dendritic spine loss from CST neurons following SCI may be due to their reduced activity. A previous study^29^ reported that pyramidal tract neurons in the M1 exhibit spontaneous discharges at a high frequency of 15 impulses/sec. Since layer V pyramidal neurons issue recurrent axon collaterals to the neighboring neurons^30^, input to their basal dendrites via the axon collaterals may generate these spontaneous discharges. Furthermore, mutual connectivity among individual motor-related areas may be involved in the morphological changes in large layer V pyramidal neurons after SCI. The SMA, PMd, and PMv are indeed interconnected with the M1^31,32^, and, therefore, the reduction in M1 activity may result in decreased activities of CST neurons in these other motor-related areas.

When the density changes post-SCI of the filopodia-, thin-, stubby-, and mushroom-type spines were investigated at the single basal dendrite level, we found that for all the motor-related areas tested, the density of filopodia-type spines was increased or tended to be increased in the SCI model, as compared to the normal control. Since this type of dendritic spine is well known as an immature type^33,34^ the spine density increase responding to SCI may not be surprising. By contrast, the other three types of spines, regarded as more mature types, were subjected to degradation related to SCI. Their density reductions were particularly evident for the M1, SMA, and PMv, whereas this dendritic event was not so distinguished or even uncertain for the PMd, which is consistent with the areal alterations in intersection number and spine density of the basal dendrites. It has been shown that the mushroom-type spine is stable and keeps its shape for a month^35^. In our experiments, the density of mushroom-type spines as well as stubby-type spines was largely decreased after SCI, especially for the M1, SMA, and PMv. This indicates that putative CST neurons (i.e., large layer V pyramidal neurons) were so drastically affected in our SCI model that even mature-type spines were subjected to degradation.

More detailed analysis of the distribution patterns of filopodia-, thin-, stubby-, and mushroom-type spines on the proximal and distal segments of basal dendrites was carried out for individual motor-related areas in the normal control and SCI model. We verified that the post-SCI increase in filopodia-type spine density was identified more prominently in the distal segments, whereas the post-SCI decrease in thin- or stubby-type spine density was seen more frequently in the proximal segments. In agreement with the results described above, the density reductions in thin-, stubby-, and mushroom-type spines were limited for the PMd, especially in the distal segments. Gordon et al.^20^ have demonstrated that dendritic spines distributed on the proximal segments display a high plasticity that is readily modified by input, whereas those on the distal segments are relatively resistant to plastic changes. It has also been documented in the same study (Gordon et al.^20^) that spine maturation may occur earlier in the distal than in the proximal segments^20^. This work strongly favors the present data indicating that mature-type dendritic spines on the proximal segments are more seriously affected by SCI, and that the emergence of immature-type dendritic spines starts from the distal segments.

Focusing on the intersection number and spine density post-SCI in the motor-related areas, no explicit or large differences were detected across the areas. In view of the fact that the basal dendrites of putative CST neurons are poorly developed for the PMd in normal macaque monkeys^18^, the limited changes in basal dendrite morphology after SCI can be plausible for the PMd. Given that CST fibers arising from the PMd terminate primarily within laminas VII and VIII of the cervical enlargement^16^, it seems likely that lesions in our SCI model may spare these laminas.

In conclusion, we have defined varying morphological alterations post-SCI of the basal dendrites of CST neurons in the motor-related areas of macaque monkeys. It is most likely that these plastic changes of CST neurons may contribute to the restoration of impaired motor functions caused by SCI at appropriate timing. Using positron emission tomography (PET) scanning, Nishimura et al. have indeed reported that the time-dependent modulation of neuronal activity during the recovery of manual dexterity from SCI differs in the M1 and PMv^36^. In our study, however, we could not follow the recovery process but observe the acute stage alone, because we applied a severely impaired SCI model who was difficult to return to normal^37^. Further investigations should be called for to clarify the roles of CST neurons in individual motor-related areas in functional restoration by using a milder SCI model.

## Materials and methods

### Animals

Four Japanese macaques (*Macaca fuscata*, 5–6 years old, 6.0–7.0 kg) of either sex were used in this study. Two of the four animals (Monkeys C and D) underwent lesions in the spinal cord as an SCI model, while the other two animals (Monkeys A and B) subserved as a normal control. The experimental protocols were approved by the Animal Welfare and Animal Care Committee of the Primate Research Institute, Kyoto University, and all experiments were conducted in accordance with the Guidelines for the Care and Use of Laboratory Primates (Ver. 3, 2010) constituted by the institute. Reporting of this study is in accordance with the ARRIVE guidelines (https://arriveguidelines.org).

### Surgical procedure

The monkeys were sedated with ketamine hydrochloride (10 mg/kg, i.m.) and xylazine hydrochloride (1 mg/kg, i.m.), and then anesthetized with 2–3% sevoflurane (Maruishi Pharmaceutical, Osaka, Japan) through a gas-administering device (Anithera-c15; Cross Medical Service, Tokyo, Japan). During the surgery, the monkeys were provided with a lactate Ringer solution (i.v.). After partial removal of the skull for ICMS mapping, a plastic chamber (67-mm long × 32-mm wide × 15-mm deep) was attached to the skull, and plastic screws were implanted into the skull as anchors. The exposed skull and screws were covered with acrylic resin (REPAIRSIN; GC, Tokyo, Japan). Finally, two head holders were mounted in parallel over the skull for head fixation.

### ICMS mapping

One week after the surgery, the head of each monkey who was seated in a primate chair was fixed to a stereotaxic frame attached to the chair. A glass-coated tungsten microelectrode (0.5–1.5 MΩ at 1 kHz; Alpha Omega, Nazareth, Israel) was inserted perpendicularly into the M1, SMA, PMd, and PMv to identify their digit representations. Parameters of stimulating currents were as follows: lower than 70 μA, 200-μs duration at 333 Hz, and trains of 11 or 44 cathodal pulses. Evoked movements were carefully monitored by muscle palpation and visual inspection, thereby preparing an ICMS map of the motor-related areas.

### SCI model

Surgical procedures for SCI were performed as previously described^37^. After ICMS mapping, the SCI model monkeys (Monkeys C and D) were again sedated with ketamine hydrochloride (10 mg/kg, i.m.) and xylazine hydrochloride (1 mg/kg, i.m.), and then anesthetized with 2–3% sevoflurane as described above. The monkeys were monitored with electrocardiogram, flow sensor, and pulse oximeter. The skin and muscles were dissected between the C4 and the Th2 segment of the spinal cord, laminectomy of the C5 to Th1 segments was performed, and the dura mater was cut unilaterally. After identification of the dorsal roots at the C6 and C7 levels, the dorsolateral funiculus was lesioned at the border between these segments using a surgical blade (No. 11) and a special needle (27G). Following sacrifice of the monkeys, spinal sections around the level of SCI were Nissl-stained with 1% Cresyl violet to confirm the lesion extent (see Suppl. Fig. 1c).

### Behavioral task

To assess manual dexterity quantitatively, we utilized a reaching and grasping task as previously described^38^. The monkeys were seated in a primate chair, and an acrylic board (14 cm × 14 cm) with three vertical (40-mm long × 22-mm wide × 10-mm deep) or horizontal (13-mm long × 40-mm wide × 10-mm deep) slots was placed in front of the chair. The monkeys were trained to reach the board and grasp a pellet (diameter, 9 mm; Osaka Maeda Seika, Osaka, Japan) from each slot within ten seconds (one trial). Data were shown as the ratio of collected pellets to the total pellets per session (21 pellets; three pellets x seven trials). We considered the trial to be successful when the monkey reached the board, grasped a pellet, and carried it into the mouth. In the SCI model, behavioral assessment was performed for 5 days before SCI and on the 10th day after SCI.

### Golgi-Cox staining

Ten days after SCI, the monkeys were perfused transcardially with 0.1 M phosphate-buffered saline (pH 7.4) under deep anesthesia with an overdose of sodium pentobarbital (50 mg/kg, i.v.). The identified digit region in each of the frontal motor-related areas was rapidly dissected out in a block and processed for Golgi-Cox staining (i.e., Golgi impregnation) according to the manufacturer’s protocol (FD Rapid GolgiStain Kit; FD NeuroTechnologies, Maryland, USA). In brief, dissected blocks containing individual motor-related areas were placed in a mixture of solutions A and B (1:1) for 2 weeks at room temperature in the dark, and the mixed solution was replaced on the next day. The blocks were then immersed in solution C for cryoprotection for 3 days at 4 °C in the dark, and the solution was replaced on the next day. Subsequently, each block was sectioned coronally at 200-μm thickness on a vibratome (Neo-LinearSlicer MT; Dosaka EM, Osaka, Japan). The sections were mounted onto gelatin-coated glass slides and reacted with a mixture of solutions D and E and distilled water (1:1:2) for 10 min at room temperature to visualize pyramidal neurons. After several washes in distilled water, the sections were dehydrated in graded alcohols, defatted in xylene, coverslipped, and then observed under a light microscope (Axio Imager Z1; ZEISS, Baden-Württemberg, Germany) with an objective lens (63 × oil, N.A 1.4, working distance 0.19 mm; ZEISS, Baden-Württemberg, Germany). Dendritic spine images were taken with Axio Imager Z1 at a resolution of 150 dpi and 2D-reconstructed by Neurolucida. Black and white reversal was done to emphasize the spine shape.

### Analyses of complexity and spine density of basal dendrites of Golgi-impregnated pyramidal neurons

The morphological analyses of basal dendrites and dendritic spines were performed as previously described with minor modifications^18^. Briefly, large layer V pyramidal neurons (i.e., putative CST neurons) in each of the motor-related areas were selected for analyses of the complexity of basal dendrites and the density of dendritic spines. In our experiments, we obtained 20 neurons from each area that possessed the somal size which meets the criteria of putative CST neurons defined in our prior study^18^ and had at least two basal dendrite arbors with multiple branching. The basal dendrites were traced by Neurolucida, and all data were incorporated into Neurolucida explorer. The complexity of the basal dendrites, comprising their total length and the number of intersections, was assessed by Sholl analysis^39^. Using this analysis, we counted the intersection number of single basal dendrites on concentric circles which start 30 µm away from the center of soma and gradually increase radii by 10 µm, and then measured the length of single basal dendrites per 10 µm. For a representative dendrite selected from each neuron, the density of dendritic spines was analyzed as the number per 10-µm dendritic segment. Since it has been described that there are only a few spines in the close vicinity of a soma^8^, we precluded the proximal segments (less than 30 µm apart from the dendritic origin) from analysis. For more detailed spine analysis, the proximal and distal segment were defined as the 30–60-µm portion from the dendritic origin and the 30-µm portion from the dendritic tip, respectively. Moreover, the dendritic spines were classified into five types by their shape^40^: filopodia type, length ≥ 2 µm, no head or head width < 0.7 µm; thin type, length: width > 1; stubby type, length: width < 1; mushroom type, head width ≥ 0.7 µm; branched type, spine head > 1. In the present work, dendritic spines of which length/width was greater than 0.2 µm were collected for the limit of spatial resolution.

### Statistical analyses

All morphological data about the basal dendrites of putative CST neurons were statistically analyzed by using R software. For Sholl analysis, the intersection number per 10-µm segment was compared by using two-way ANOVA with the Tukey–Kramer method. Comparison of the dendritic spine density between the normal control and the SCI model was done each motor-related area by using the two-way ANOVA with the Bonferroni post hoc test. The density of each spine type in the SCI model was compared among the motor-related areas by using one-way ANOVA with the Tukey–Kramer method. The statistical significance was accepted at *p* < 0.05.

## Supplementary Information

Supplementary Figure 1.

Supplementary Information 2.

## Data Availability

The datasets of large layer V pyramidal neurons analyzed during the current study are available from the corresponding author upon reasonable request. Source data are provided with this paper.
