# Supplementary material for: Morphological changes of large layer V pyramidal neurons in cortical motor-related areas after spinal cord injury in macaque monkeys
Source: Sci Rep. 2023 Jan 3;13:82. doi: 10.1038/s41598-022-26931-3 (PMC9810718; doi:10.1038/s41598-022-26931-3)
Supplement: Supplementary file 1 — Supplementary Figure 1. [file 41598_2022_26931_MOESM1_ESM.pdf]

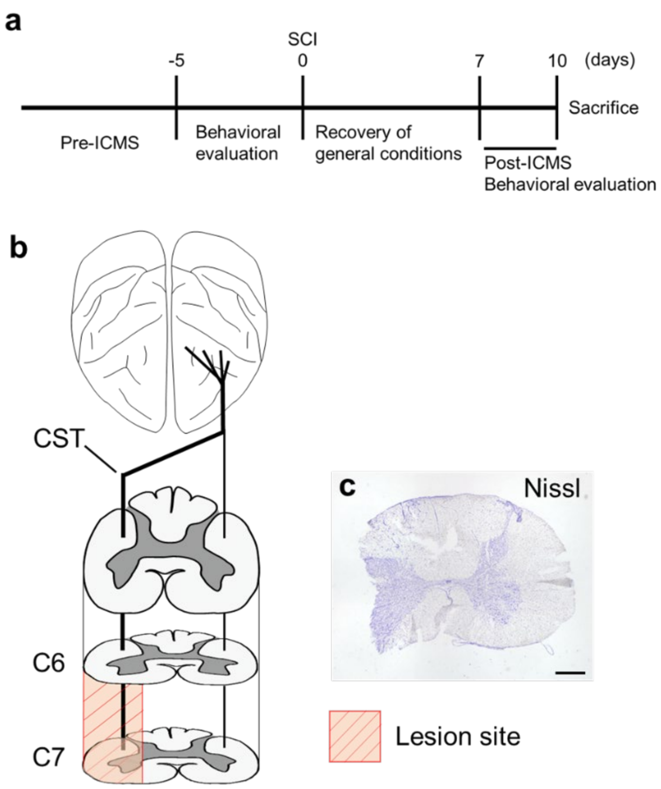

Supplementary Figure 1. Time-course and extent of SCI

(a) Time-course of experiments. (b) Unilateral SCI between the C6 and the C7 segment of the cervical enlargement. (c) Nissl-stained transverse section at the level of SCI. Scale bar, 1 mm.
